# Microengineered Multi‐Organoid System from hiPSCs to Recapitulate Human Liver‐Islet Axis in Normal and Type 2 Diabetes

**DOI:** 10.1002/advs.202103495

**Published:** 2021-12-23

**Authors:** Tingting Tao, Pengwei Deng, Yaqing Wang, Xu Zhang, Yaqiong Guo, Wenwen Chen, Jianhua Qin

**Affiliations:** ^1^ Division of Biotechnology Dalian Institute of Chemical Physics Chinese Academy of Sciences Dalian 116023 China; ^2^ Institute for Stem Cell and Regeneration Chinese Academy of Sciences Beijing 100101 China; ^3^ CAS Center for Excellence in Brain Science and Intelligence Technology Chinese Academy of Sciences Shanghai 200031 China; ^4^ University of Chinese Academy of Sciences Beijing 100049 China

**Keywords:** hiPSCs, liver, multi‐organoid‐on‐chip, pancreatic islet

## Abstract

Type 2 diabetes mellitus (T2DM) is a systematic multi‐organ metabolic disease, which is characterized by the dynamic interplay among different organs. The increasing incidence of T2DM reflects an urgent need for the development of in vitro human‐relevant models for disease study and drug therapy. Here, a new microfluidic multi‐organoid system is developed that recapitulates the human liver‐pancreatic islet axis in normal and disease states. The system contains two compartmentalized regions connected by a microchannel network, enabling 3D co‐culture of human induced pluripotent stem cells (hiPSC)‐derived liver and islet organoids for up to 30 days under circulatory perfusion conditions. The co‐cultured liver and islet organoids exhibit favorable growth and improved tissue‐specific functions. Transcriptional analyses reveal the activation of metabolically relevant signaling pathways in the co‐cultured organoids. Notably, the co‐culture system facilitates sensitive glucose‐stimulated insulin secretion from islet organoids and increased glucose utilization in liver organoids by glucose tolerance tests. Both liver and islet organoids display mitochondrial dysfunction and decreased glucose transport capacity under high glucose conditions, which can be alleviated by metformin treatment. This novel multi‐organoid system can recapitulate human‐relevant liver‐islet axis under both physiological and pathological conditions, providing a unique platform for future T2DM research and drug development.

## Graphical abstract

Type 2 diabetes mellitus (T2DM) is a systematic metabolic disease, which is characterized by the dynamic interplay among different organs. Here, a new microfluidic multi‐organoid system is developed from human induced pluripotent stem cells to recapitulate the liver‐pancreatic islet axis in normal and disease states. It provides a unique platform for T2DM research and drug development.

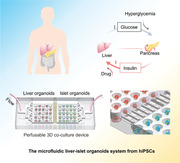

## 1 Introduction

In recent decades, the epidemic of type 2 diabetes (T2DM) has come to represent one of the most pressing and costly medical challenges facing modern society.^[^ ^1^, ^2^, ^3^, ^4^ ^]^ T2DM is characterized by hyperglycemia and insulin resistance, which is commonly accompanied by a combination of pancreatic *β*‐cell dysfunction and insulin resistance in the liver. In vivo, glucose homeostasis is crucial for regulating metabolism and meeting the energy requirements of individuals. Blood glucose levels are governed by the collective effort of multiple tissues, including the liver, pancreatic islets, adipose, and muscle.^[^ ^5^, ^6^, ^7^ ^]^ For example, the liver and pancreatic islets show a dual function in maintaining euglycemia. In the fasted state, pancreatic islet *α*‐cells secret glucagon, and hepatic glucose production is induced to supply energy to vital organs. Postprandially, pancreatic islet *β*‐cells secret insulin to induce hepatic glucose uptake and facilitate glucose breakdown.^[^ ^8^, ^9^, ^10^ ^]^ Failure of these coordinated inter‐organ interactions may lead to dysregulated glucose levels and metabolic disorders, such as T2DM.

Current efforts to study glucose metabolism and T2DM have largely relied on animal models.^[^ ^11^, ^12^ ^]^ However, animal models do not accurately represent human physiology due to significant species‐specific differences and thus lack the ability to model human metabolic responses. In particular, due to the coordinated inter‐organ effects of systemic factors, such as hormones and nutrients, distinguishing direct and indirect effects on the organ systems in vivo remains challenging.^[^ ^13^, ^14^, ^15^ ^]^ In addition, studies of the interplay between different tissues in vitro have thus far been limited to cell co‐cultures. Previous studies have used a mixture of primary animal hepatocytes and pancreatic islet *β*‐cells or cell lines.^[^ ^16^, ^17^, ^18^ ^]^ Although these methods allow the routine interrogation of cellular crosstalk, they do not precisely control the human organ‐specific features and accurately recapitulate in vivo‐like signaling feedback loop.

Organoids represent a new class of 3D tissues that can recapitulate the key structural and functional aspects of their in vivo counterparts.^[^ ^19^, ^20^, ^21^ ^]^ Generally, organoids are generated by self‐organization of pluripotent stem cells (PSCs) and guided through various developmental processes to allow further lineage differentiation. To date, a variety of human organoids (e.g., brain,^[^ ^22^ ^]^ liver,^[^ ^23^ ^]^ intestine,^[^ ^24^ ^]^ and islet^[^ ^25^ ^]^) have been successfully established and show great potential for the study of organ development, disease modeling, and drug screening. Recently, organoids‐on‐a‐chip technologies have opened up new avenues for creating high fidelity organ models by combining developmental biology principles with microfluidic chip technology.^[^ ^26^, ^27^, ^28^, ^29^, ^30^, ^31^ ^]^ Compared to conventional methods, organoids‐on‐a‐chip systems offer a controlled tissue microenvironment by providing accurate biomechanical cues, biochemical signals, and organ‐organ interactions.^[^ ^4^, ^32^, ^33^, ^34^ ^]^ Currently, several organoids‐on‐a‐chip systems have been established, including islet, liver, heart, and brain, and have been used for environmental toxicity screening, drug testing, and studying organ‐organ interactions.^[^ ^35^, ^36^, ^37^, ^38^ ^]^

In this study, we present a microfluidic multi‐organoid system for studying the human liver‐pancreatic islet axis in terms of insulin and glucose regulation in normal and disease settings. The human induced pluripotent stem cells (hiPSC)‐derived liver and islet organoids were co‐cultured for up to one month in a circulatory perfusion system. Cell viability and organ‐specific functions of the liver and islet organoids were examined under mono‐ and co‐culture conditions. Dynamic interplay between the organoids was observed, reflected in glucose‐stimulated insulin secretion (GSIS) from the islet organoids and altered glucose utilization in the liver organoids. Especially, we evaluated the effects of high glucose (25 mm) on mitochondrial and glucose transport function in this system, as well as the effects of the anti‐diabetic drug metformin thereon. This newly established approach provides a promising and robust in vitro multi‐organoid system for studying the complex pathogenesis of T2DM and will allow the development of novel therapeutics.

## 2 Results

### 2.1 Design and Operation of the Microfluidic Multi‐Organoid System

**Figure 1:**
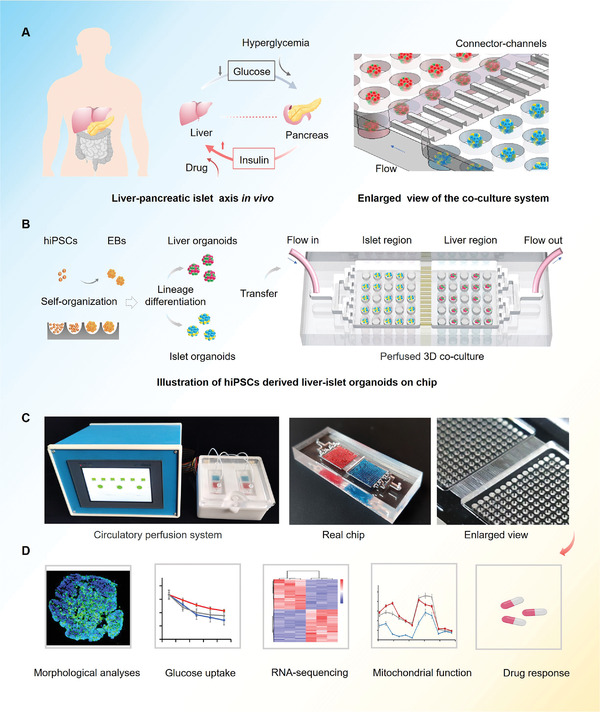
Schematic of hiPSCs derived multi‐organoid‐on‐chip system to model human liver‐pancreatic islet axis in vitro. A) Simplified illustration of the crosstalk between human liver and islet tissues during normal glucose regulation in vivo. B) Procedures of the differentiation and generation of hiPSCs derived liver and islet organoids in the micro‐well device and co‐culture experimental set‐up in the multi‐organoid chip. The device consists of two culture compartments connected by parallel network to support the co‐culture of two types of organoids. C) Experimental setup of the microfluidic perfusion system and the real chip. D) Functional analysis of the liver‐islet organoids system was carried out using several experimental methods.

In vivo, insulin secreted from pancreatic islet *β*‐cells promotes hepatic glucose uptake to maintain blood glucose levels within the normal range. This process represents the dynamic interplay between the human liver and pancreatic islet cells, as shown in **Figure** **1**A. To recapitulate the internal glucose regulation between liver and islet in vivo, we built a microfluidic multi‐organoid system including an organoid co‐culture chip, peristaltic pump, and perfusion device. The microfluidic chip consisted of two compartments connected by a parallel microchannel network. Each compartment contained an array of microwells to maintain the 3D culture of liver and islet organoids. The interconnecting microchannels facilitated the exchange of media and secreted metabolic products between two types of organoids. This approach the enabled co‐culture of the liver and islet organoids in a perfusable chip system using a one‐step method that was more convenient to operate.

The liver and pancreatic islet organoids were generated by self‐organization of hiPSCs in the microwell arrays, which rely on lineage‐specific differentiation protocols. As a result of the microwell design, embryoid bodies (EBs) with uniform size (200 µm in diameter) and morphology were formed, resulting in reduced variability of the organoids generated. By adding defined growth factors and small molecules to the culture media, the EBs were differentiated into endoderm cells, followed by liver or pancreatic lineages at days 20 and 23, respectively. Then, the liver and islet organoids were transferred into the chip device for long‐term co‐culture (Figure 1B). To ensure sufficient insulin secretion from islet organoids and to moderate glucose consumption by the liver organoids, the organoids were seeded in the respective chambers at a 1:1 ratio. A peristaltic pump was used to provide a circulatory media flow (100 µL h^−1^), thus providing a dynamic microenvironment for organoids during long‐term culture (Figure 1C). The functional features of the co‐cultured liver and islet organoids in the chip system are analyzed using different methods (Figure 1D).

### 2.2 Characterization of the hiPSC‐Derived Liver and Pancreatic Islet Organoids

**Figure 2:**
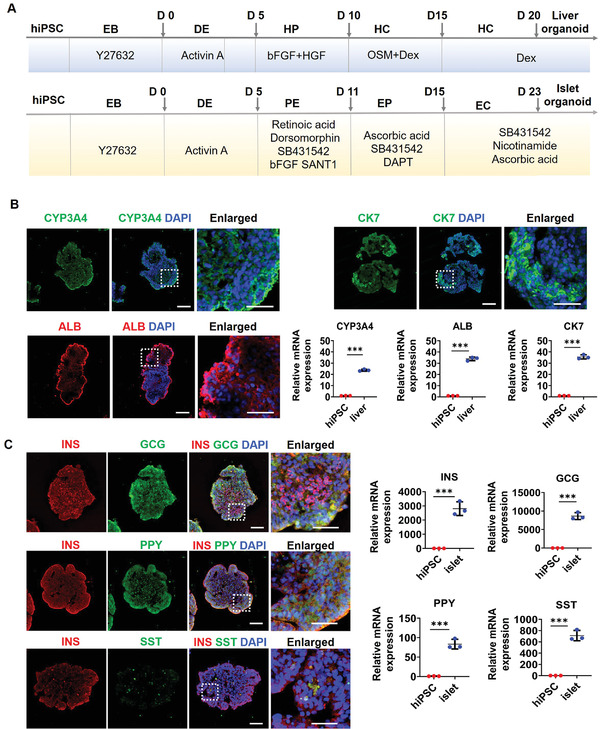
Identification of organ‐specific protein and gene expressions in liver and islet organoids prior to co‐culture assay. A) Timeline illustrating the differentiation process for liver and islet organoids from hiPSCs. DE, definitive endoderm; HP, hepatic progenitor; HC hepatocyte; PE, pancreatic endoderm; EP, endocrine progenitor; EC, endocrine cell. B) Immunohistochemical staining of hepatic‐specific proteins in liver organoids at day 20, including cytochrome P450 (CYP3A4), hepatocyte (ALB), and cholangiocyte (CK7). Quantification of relative mRNA expression levels of *CYP3A4*, *ALB_,_* and *CK7* genes in liver organoids using RT‐PCR. C) The expression of pancreatic‐lineage markers for *β*‐cells (INS), *α*‐cells (GCG), *γ*‐cells (PPY), and *δ*‐cells (SST) in islet organoids at day 23 were determined by immunofluorescence staining. Quantification of relative mRNA expression levels of *INS*, *GCG*, *PPY_,_* and *SST* genes in islet organoids using RT‐PCR. Nuclei were stained with DAPI (blue). Scale bars: 100 µm. Enlarged images indicated by the white squares are shown on the right. Scale bars: 20 µm. Expression values were normalized to GAPDH. Relative expression is represented as the mean ± SEM, three independent experiments were performed. The data were analyzed using Student's t‐test, *p < 0.05, **p < 0.01, and ***p < 0.001.

HiPSCs derived organoids contain the typically cellular component and functional properties of their internal parts, which holds a robust power for studying human organogenesis development and disease.^[^ ^39^, ^40^, ^41^ ^]^ But the heterogeneity of PSCs generated organoids limited their application of biomedical research.^[^ ^42^, ^43^ ^]^ Previous study has demonstrated that the morphology of organoids may relate to the lineage‐specific differentiation of stem cells and affect the cell viability of organoids.^[^ ^44^, ^45^ ^]^ For this point, we used a size‐optimized high‐throughput microwell chip for the controllable formation of EBs with uniform size and morphology, which is facilitated to reduce the variability of the generated liver and isle organoids on the device. Prior to the co‐culture of the liver and pancreatic islet organoids on a chip, we initially generated the two organoids from hiPSCs in microwell arrays as described in **Figure** **2**A. The differentiation process for the hepatic lineage included four stages: definitive endoderm (DE), hepatic endoderm (HE), hepatic progenitor (HP), and HC. Similarly, pancreatic endocrine lineage differentiation also involved four stages: DE, pancreatic endoderm (PE), endocrine progenitor (EP), and endocrine cell (EC). The organoids were generated from hiPSCs in a polydimethylsiloxane (PDMS) microwell chip by adding various growth factors or small molecules to the defined media at distinct stages of differentiation. The morphology and size distributions of the liver and islet organoids were observed on days 0, 5, 11, 20, and 23. The data showed that the average sizes of both liver and islet organoids gradually increased during the differentiation process (Figure S1A–C, Supporting Information). Additionally, cell viability was assessed by live/dead staining and flow cytometry assays. A high proportion of the liver (≈99.03%) and islet (≈96.11%) organoid cells were viable (Figure S1D,E, Supporting Information), indicating that the liver and islet organoids maintained favorable morphology and cell viability during development.

To further validate the functionality of the liver organoids, we identified liver‐specific cell types and protein secretion using immunostaining and real‐time polymerase chain reaction (RT‐PCR) on day 20. As shown in Figure 2B, the liver organoids expressed high levels of a cytochrome P450 marker (CYP3A4) related to the metabolic capacity of the liver. Additionally, mature hepatocyte (ALB) and cholangiocyte (CK7) markers were highly expressed, revealing cellular heterogeneity within the liver organoids. Consistently, the RT‐PCR data showed high expression of *CYP3A4, ALB*, and *CK7* genes in the liver organoids. Meanwhile, the mRNA expression of *CYP3A7*, a fetal counterpart of *CYP3A4*, was significantly up‐regulated in liver organoids indicating the immature phenotype of hiPSCs derived liver organoids (Figure S5, Supporting Information). Overall, these results demonstrate that the hiPSC‐derived fetal liver organoids possess the ability to metabolize drugs and contain key cellular components of the native liver.

Similarly, the differentiation of hiPSC‐derived islet organoids was confirmed by immunohistochemical analysis and RT‐PCR. In vivo, pancreatic islets are multicellular tissues containing four types of ECs: pancreatic *β*‐, *α*‐, *γ*‐, and *δ*‐cells. These cell types are essential for regulating blood glucose homeostasis. As shown in Figure 2C, specific markers of *β*‐cells (insulin, INS), *α*‐cells (glucagon, GCG), *γ*‐cells (pancreatic polypeptide, PPY), and *δ*‐cells (somatostatin, SST) were abundantly expressed in the islet organoids by day 23. Consistently, RT‐PCR data revealed that the expression levels of pancreatic islet‐specific genes were upregulated by day 23. These results suggest that the heterogeneous islet organoids generated in this study contain the four classical EC types, making them analogous to human pancreatic islets.

### 2.3 Improved Function of Co‐Cultured Liver and Islet Organoids on a Chip

**Figure 3:**
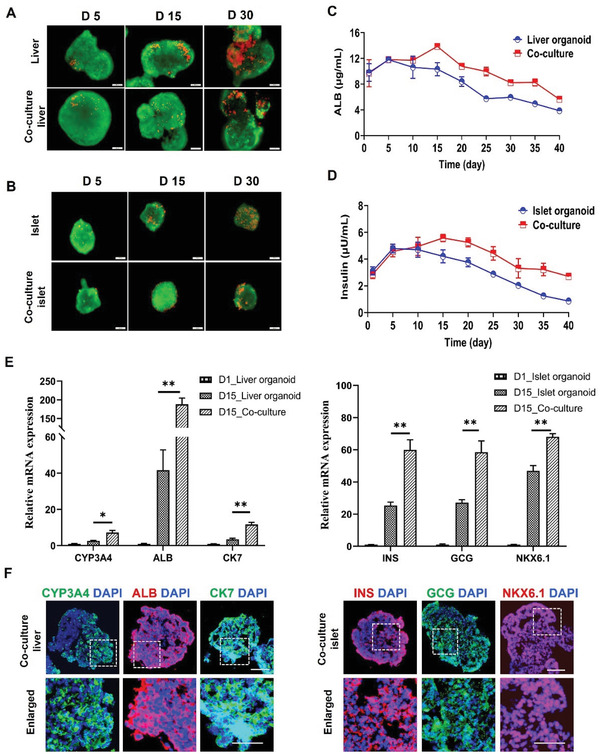
Comparison of cell viability and functionality of liver and islet organoids under mono‐ and co‐culture conditions. A,B) Liver and islet organoids were stained with a live/dead kit on days 5, 15, and 30 during the prolonged culture period. C,D) Examination of albumin secretion by liver organoids and insulin secretion by islet organoids under different culture conditions from days 1 to 40. E) Quantification of relative mRNA expression levels of liver‐specific genes (*CYP3A4*, *ALB*, and *CK7*) and islet‐associated genes (*NKX6*.1, *INS*, and *GCG*) in organoids at day 15 using RT‐PCR in mono‐ and co‐culture groups. Expression values were normalized to GAPDH. Relative expression is represented as the mean ± SEM, three independent experiments were performed. The data were analyzed using Student's t‐test, *p < 0.05, **p < 0.01, and ***p < 0.001. F) Immunohistochemical staining of CYP3A4 (red), ALB (red), and CK7 (green) in liver organoids and NKX6.1 (red), INS (red), and GCG (green) in islet organoids at day 15. DAPI was used to stain the nuclei (blue). Scale bars: 100 µm. Enlarged views are shown below each image. Scale bars: 20 µm.

To investigate the long‐term functionality of co‐cultured liver and islet organoids on a chip, we assessed cell viability in mono‐ and co‐cultures. The liver and islet organoids were transferred to the chip device and cultured in a co‐culture medium. During the culture period of 30 days, the liver organoids showed a higher cell viability in co‐culture compared with mono‐culture (Figure **3**A). Similarly, islet organoids showed improved survival in co‐culture (Figure 3B). These results indicate that the multi‐organoid system facilitates the growth and cell viability of organoids during a prolonged culture period. Next, we examined albumin synthesis in the liver organoids under mono‐ or co‐culture conditions. It appeared that albumin secretion increased gradually before day 15, then slowly decreased until day 40. Notably, the 15‐day liver organoids in co‐culture displayed a higher level (≈14 µg mL^−1^) of albumin secretion than that of those in mono‐culture (≈10 µg mL^−1^). Moreover, the co‐cultured liver organoids maintained albumin production (9.8 µg mL^−1^) at day 25, while this was not seen in mono‐culture. These results demonstrated that the co‐culture system improved the hepatic‐specific function when compared with mono‐culture (Figure 3C). Additionally, insulin secretion by the islet organoids in response to glucose exposure was examined. Similarly, insulin secretion increased gradually prior to day 15 and subsequently decreased until day 40. The islet organoids displayed enhanced insulin secretion function in the co‐culture system compared with those in mono‐culture during the long‐term culture period (Figure 3D).

To determine the expression of organ‐specific markers in the liver and pancreatic islet organoids under mono‐ and co‐culture conditions, we further performed RT‐PCR and immunofluorescence analysis. The gene expression profiles of hepatic‐lineage specific and CYP450 genes were quantified in the liver organoids on days 5, 15, and 30 under different culture conditions. The expression levels of *ALB, CK7*, and *CYP3A4* were significantly upregulated in co‐cultured organoids at day 15 (Figure 3E). However, these genes were not significantly different between mono‐ and co‐culture systems at days 5 or 30 (Figures S2A and S3A, Supporting Information). Meanwhile, the distribution and expression of the corresponding proteins (CYP3A4, ALB, and CK7) were evaluated in liver organoids on days 5, 15, and 30 under co‐culture conditions. Immunofluorescence results showed significant expression of CYP3A4, ALB, and CK7 proteins in the co‐cultured liver organoids at day 15 (Figure 3F, Figures S2B and S3B, Supporting Information), consistent with the gene expression profiles. These results indicated that the expression of functional genes in liver organoids was enhanced through co‐culture with islet organoids, and this enhancement occurred following an appropriate time in co‐culture (≈day 15). For islet organoids, pancreas‐associated genes were examined in mono‐ and co‐cultures at different time points (days 5, 15, and 30), including a *β*‐cell maturation‐associated transcription factor (*NKX6.1*), *INS*, and *GCG*. These genes showed higher expression in co‐cultured islet organoids compared with those in mono‐culture at days 5 and 15, but no significant difference was seen at day 30 (Figure 3E, Figures S2A and S3A, Supporting Information). Moreover, NKX6.1, INS, and GCG proteins were abundant in islet organoids at days 5 and 15 (Figure 3F, Figure S2B, Supporting Information). These data confirmed that our co‐culture system facilitated pancreatic lineage differentiation and EC maturation in islet organoids.

Interestingly, previous studies have demonstrated that both liver and islet appear to benefit each other by the secreted hormones, cytokine, and micro‐RNAs in vivo, including insulin, GLP‐1, and microRNA‐26a.^[^ ^46^, ^47^ ^]^ Based on a defined co‐culture medium without complex growth factors, this dynamic co‐culture system could facilitate the exchange of these extracellular factors between liver and islet organoids. This multi‐organoid‐chip system could enable to recapitulate the cross‐talk between liver and islet in vitro and promote the biological functions including efficient differentiation, favorable cell viability, and tissue‐specific functions of co‐cultured organoids for extended durations. The established chip system provides a robust platform for studying bidirectional signaling between liver and islet cells.

### 2.4 Transcriptional Analysis of Co‐Cultured Liver and Islet Organoids

**Figure 4:**
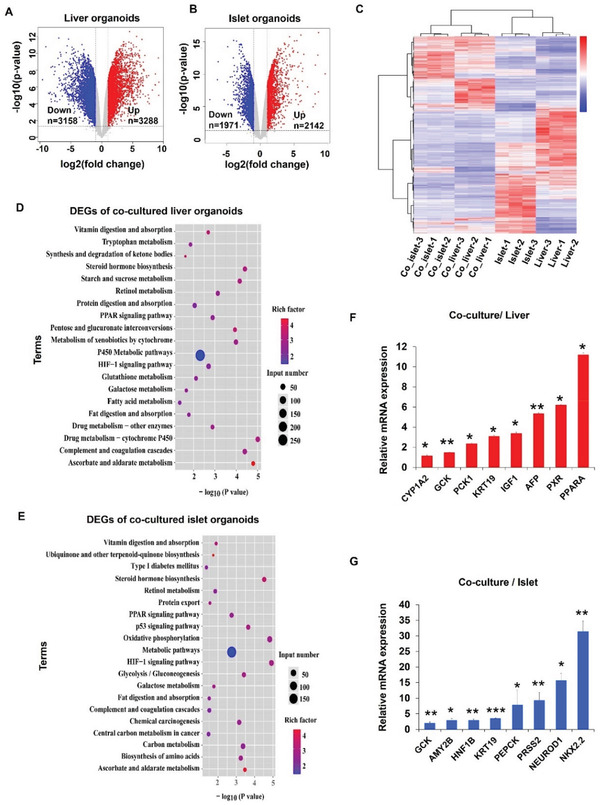
Transcriptomic analysis of liver and islet organoids in the multi‐organoid‐on‐chip system at day 15. A,B) Volcano plots of the significantly DEGs in liver and islet organoids in co‐cultures compared to mono‐cultures. Genes differentially expressed with fold change over 2.0 and *p*<0.05 were marked in color. *P* values were calculated using a two‐sided, unpaired student's t‐test with equal variance assumed (n = 3 independent biological samples). C) Hierarchical clustering heat map for DEGs between mono‐ and co‐cultured liver and islet organoids. Three repeats were performed. The gradient color scale at the right top indicates the log_2_(fold change) in the expression of the treatment case compared with the control case. D,E) KEGG functional classification of the liver and islet organoid DEGs under mono‐ and co‐culture conditions at day 15. The color of the dots represents the rich factor, while the size represents the input number of genes for each KEGG term. The horizontal axis indicates the significance of enrichment (−log_10_ (*p*‐value)). F,G) Validation of selected DEGs identified by RNA‐seq using qRT‐PCR. The expression values were normalized to GAPDH. Data were normalized against mono‐culture expression values and are shown as mean ± SEM, three independent experiments were performed.

To understand the transcriptional responses of the islet and liver organoids in co‐culture, we performed RNA sequencing (RNA‐seq) analysis of islet and liver organoids at day 15 under mono‐ and co‐culture conditions. A total of 6446 differentially expressed genes (DEGs) were identified in the co‐cultured liver organoids, including 3158 upregulated and 3288 downregulated genes (**Figure** **4**A). Additionally, 4113 DEGs (1971 upregulated and 2142 downregulated genes) were identified in the co‐cultured islet organoids (Figure 4B). Hierarchical clustering of these genes demonstrated significant differences between organoids in mono‐culture and co‐culture systems (Figure 4C), revealing that both islet and liver organoids were greatly affected by the co‐culture system.

To further investigate the responses of the co‐cultured organoids to cooperative signaling between the organoids, Kyoto Encyclopedia of Genes and Genomes (KEGG) enrichment analysis was performed to identify specific biological processes enriched among the significantly upregulated genes. Interactions between the islet and liver organoids enhanced several pathways compared with isolated organoids. These pathways included CYP450 metabolism, fatty acid metabolism, and peroxisome proliferator‐activated receptors (PPARs) signaling in the liver organoids, all of which are crucial for regulating drug metabolic activity, lipid metabolism, insulin sensitivity, and liver homeostasis (Figure 4D). Moreover, agonists of several PPAR isoforms have been used for T2DM treatment,^[^ ^48^ ^]^ indicating that PPARs may serve as potential therapeutic targets for other metabolic diseases. In comparison, pathways related to metabolism, glycolysis/gluconeogenesis, and protein digestion and absorption were upregulated in the islet organoids during co‐culture (Figure 4E). Glucose metabolic pathways are vital for GSIS.^[^ ^49^ ^]^ These results indicated that the multi‐organoid system could recapitulate the functional coupling of human liver and islet cells.

To quantify the expression of functional and metabolism‐dependent genes among the upregulated genes associated with significantly regulated biological processes in the co‐cultured liver and islet organoid, RT‐PCR assay was performed. In the liver organoids, transcription of hepatic‐specific genes (*AFP, KRT19, PXR*), a growth and metabolism regulator gene (*IGF1*), lipid and glucose metabolism‐related genes (*PPARA, GCK, PCK1*), and a CYP450‐associated gene (*CYP1A2*) were significantly upregulated (Figure 4F) but the apoptosis‐related gene (CASP9) were significantly down‐regulated in co‐culture group (Figure S4A, Supporting Information). Similarly, genes relating to pancreatic *β*‐cell maturation (*NKX2.2, HNF1B, NEUROD1*), pancreatic exocrine cell function (*AMY2B, PRSS2, KRT19*), and lipid and glucose metabolism (*PEPCK, GCK*) were significantly upregulated in the islet organoids (Figure 4G), but the apoptosis‐related genes (CASP6, CASP9) were significantly down‐regulated in co‐cultured islet organoids (Figure S4B, Supporting Information). Collectively, these data revealed the diverse responses of the liver and islet organoids in a complex setting, which may provide new insights into the role of liver‐islet cross‐talk in cell viability, secretion functions and phenotype expression, and glucose homeostasis.

### 2.5 Crosstalk Between Liver and Islet Organoids on Chip

**Figure 5:**
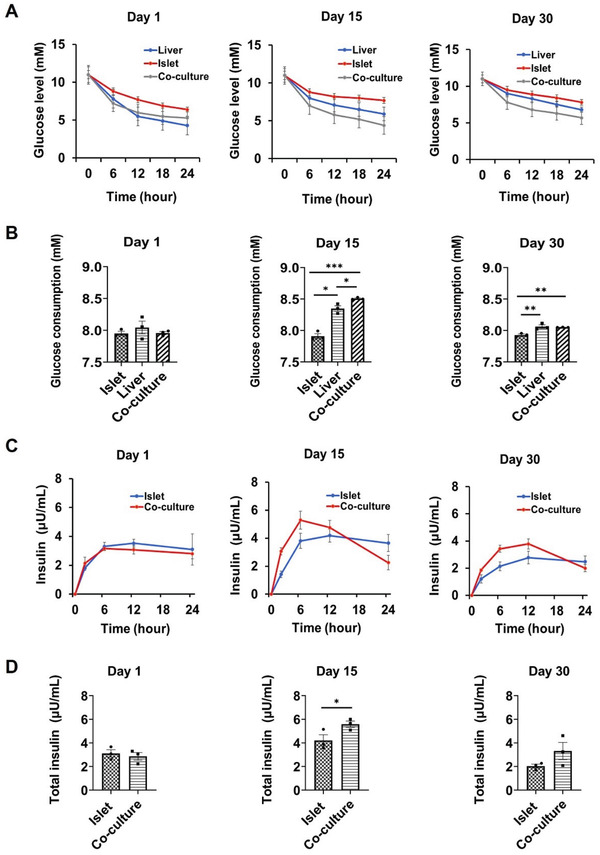
Characterization of the interplay between liver and islet organoids in a perfused multi‐organoid chip system. A) Dynamic glucose levels were quantified using a GTT at days 1, 15, and 30 in mono‐ and co‐cultures. Medium containing 11 mm glucose was injected into the mono‐ or co‐culture systems. B) Area under the glucose consumption curve on days 1, 15, and 30. C) Insulin secretion by pancreatic organoids during GTT under different conditions on days 1, 15, and 30. D) Area under the insulin response curve on days 1, 15, and 30. The data were analyzed using Student's t‐test or a one‐way analysis of variance (ANOVA) test and are shown as the mean ± SEM, three independent experiments were performed. *p < 0.05, **p < 0.01, and ***p < 0.001.

In the human body, pancreatic islets secrete insulin to promote glucose uptake by the liver from the bloodstream. A standard oral glucose tolerance test (GTT) is one of the most commonly used clinical methods to assess glucose utilization in the body.^[^ ^50^ ^]^ Therefore, to assess possible interactions between the islet and liver organoids in this microfluidic chip system, we performed a surrogate GTT assay to evaluate glucose regulation. The glucose level in the co‐culture medium was adjusted to 11 mm by a complete media exchange to simulate postprandial glucose levels. To assess glucose utilization by the liver tissue, glucose levels were quantified in the supernatants of liver organoid monoculture, islet organoid monoculture, and co‐culture conditions at different time points. The supernatant glucose concentration was decreased from 11 mm to within the normal range (3.9–6.1 mm) and showed a more sensitive blood glucose regulation process in co‐cultures within 24 h on days 1, 15. However, the mono‐cultured islet organoid group remained a hyperglycemic level. The mono‐cultured liver could reduce the glucose concentration to a normal range, but this response is not sensitive due to the lack of insulin stimulation (**Figure** **5**A). Glucose utilization was estimated by calculating the area under the glucose response curve. Compared with the mono‐cultures, glucose consumption was significantly increased in the co‐culture system on day 15 (Figure 5B). These results indicated that the liver‐islet organoids‐on‐a‐chip system could recapitulate insulin‐stimulated glucose uptake and maintenance the ability of physiological postprandial blood glucose regulation in vitro.

A key functional feature of pancreatic *β*‐cells is the ability to time‐efficiently perform GSIS. Previous study has proved that the function of hiPSC‐derived pancreatic *β*‐cells is similar to primary human *β* cells.^[^ ^51^ ^]^ The levels of secreted insulin in the circulating medium were examined after exposure to 11 mm glucose in mono‐ and co‐cultures at days 1, 15, and 30. As shown in Figure 5C, insulin levels rose steadily in the co‐culture condition in response to glucose, and reached a steady‐state once glucose levels had dropped to normal levels at days 1, 15, and 30. These results indicated that the co‐cultured islet organoids were sensitive to glucose stimulation. Significantly increased insulin secretion was seen in the co‐culture system within 24 h of glucose stimulation compared to the mono‐cultures, particularly on day 15 (Figure 5D). It appeared that the co‐culture system facilitated the ability of islet organoids to sustain insulin secretion in hyperglycemic conditions, leading to the recovery of glucose concentrations to normal levels. Overall, these results confirmed that the established multi‐organoid‐on‐chip system could model the regulation of glucose by pancreatic *β*‐cell‐derived insulin efficiently and stimulate hepatic glucose uptake. This novel platform provides a robust method to study the feedback loop within the liver‐islet axis that maintains glucose levels within a normoglycemic range in vitro.

### 2.6 Response of the Multi‐Organoid System to Hyperglycemic Conditions

**Figure 6:**
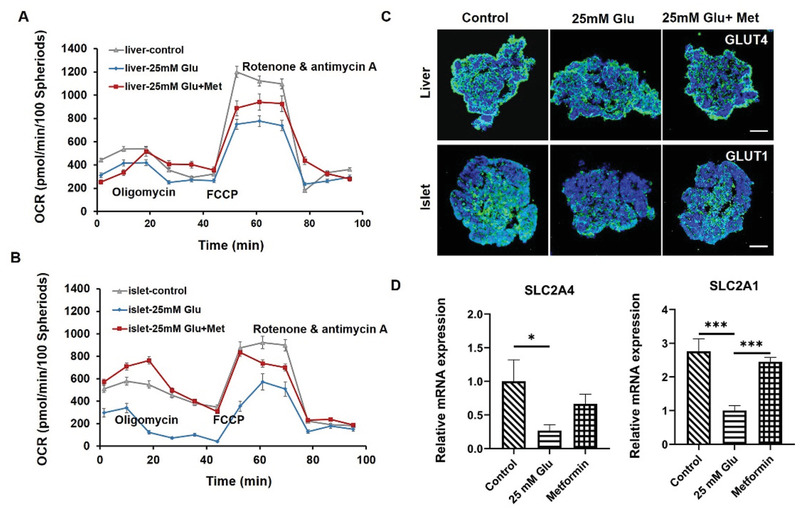
Assessment of mitochondrial function and glucose transport ability in the islet‐liver organoids system in response to hyperglycemia condition and metformin treatment. A,B) Liver and islet organoids were co‐cultured for 15 days and then exposed to three culture conditions (control, 25 mm glucose, and 25 mm glucose with 100 mm metformin). OCR of the liver and islet organoids under these three conditions were measured after 5 days. Oligomycin, FCCP, and rotenone/antimycin A were added at the indicated time points during the experiments, and the data were normalized to cell numbers. C) Immunofluorescence staining of the glucose transport protein marker GLUT1 in islet organoids and GLUT4 in liver organoids in co‐culture under different treatment conditions at day 15. Scale bars: 100 µm. D) Quantitative analysis of relative mRNA expression levels of *SLC2A1* in islet organoids and *SLC2A4* in liver organoids using RT‐PCR at day 15. The data were analyzed using a one‐way ANOVA with the Bonferroni post‐hoc test and are presented as the mean ± SEM, three independent experiments were performed, *p < 0.05, **p < 0.01, and ***p < 0.001.

Chronic hyperglycemia is a leading cause of T2DM and involves multiple metabolically functional organs, including the liver and pancreatic islet cells. To evaluate mitochondrial function and glucose transporter expression in the co‐cultured liver and islet organoids in a hyperglycemic environment, the multi‐organoid chip system was exposed to media containing a high concentration of glucose (25 mm) for 5 days. It has been previously reported that mitochondria impairment can lead to T2DM.^[^ ^52^, ^53^, ^54^ ^]^ We evaluated mitochondrial function by quantifying the cellular oxygen consumption rate (OCR). The hyperglycemic condition reduced spare respiratory capacity and suppressed ATP production in both liver and islet organoids (**Figure** **6**A,B). These results indicate that mitochondrial function can be weakened by hyperglycemic conditions in this in vitro system.

Glucose transporters (GLUT1, GLUT3, and GLUT4) are specific membrane proteins that can transport extracellular glucose into cells and alter cellular glucose uptake and metabolism.^[^ ^55^, ^56^ ^]^ We thus evaluated the protein abundance and gene expression of GLUT1 in islet organoids and GLUT4 in liver organoids under hyperglycemic conditions. GLUT1 and GLUT4 proteins were significantly reduced in abundance in the hyperglycemic islet and liver organoids, respectively (Figure 6C). Consistently, the expression levels of *SLC2A4* (encoding GLUT4) and *SLC2A1* (encoding GLUT1) were reduced in organoids under hyperglycemic conditions (Figure 6D). These results demonstrated that the expression levels of glucose transporter proteins were reduced in the liver and islet organoids in a hyperglycemic environment, simulating the pathological processes known to occur in the early stage of diabetes. Based on these results, the liver and pancreatic islet multi‐organoid system can recapitulate the liver‐islet axis and display the pathological features of T2DM when challenged with a high glucose level.

Metformin is a drug widely used in the treatment of diabetes, which could affect the mitochondrion and cell glucose transporter proteins of cells for the regulation of cellular glucose metabolism.^[^ ^57^ ^]^ To further evaluate the multi‐organoid system in response to drug treatment, the organoids were exposed to a high‐glucose (25 mm) culture medium containing metformin (100 mm). The results revealed improved respiratory capacity and ATP production in the liver and islet organoids following metformin treatment, indicating the ability of metformin to restore mitochondrial function (Figure 6A,B). Additionally, the expression of glucose transporter proteins (GLUT1 and GLUT4) and genes (*SLC2A1*and *SLC2A4*) were significantly increased in the islet and liver organoids, reflecting restored glucose transport functions in response to metformin treatment (Figure 6C,D). Overall, these results reflect the potential for the proposed liver‐islet organoid system to be used for drug screening for novel treatments for T2DM in a multi‐organ manner.

## 3 Discussion

T2DM is a systematic multi‐organ metabolic disease. Glucose homeostasis is essential for maintaining human health, and is coordinated by dynamic interplay between different organs in vivo. Disruption of this coordination can lead to hyperglycemia and metabolic disorders, such as diabetes mellitus. The liver and pancreas are two key organs involved in physiological glucose homeostasis, the crosstalk between them greatly contributes to the normal regulation of blood glucose levels. Here, we present a multi‐organoid‐on‐chip system derived from hiPSCs to model the liver‐pancreatic islet axis in vitro and to investigate the crosstalk between liver and islet in normal and type 2 diabetes.

The liver‐islet multi‐organoid system established in this study enabled the interrogation of organ‐organ interactions under perfused co‐culture conditions for up to 30 days. GTT is the clinical research method for evaluating glucose utilization in humans.^[^ ^50^ ^]^ A surrogate functional GTT assay in this system demonstrated that the liver‐islet interplay in glucose regulation mainly involves the insulin secretion from the islet organoids and glucose uptake by the liver organoids. The islet organoids secreted insulin in response to glucose and promoted hepatic glucose uptake in the co‐culture system. Moreover, the glucose levels decreased more faster in the co‐culture system and returned to normal fasting levels after 24 h, which was not observed in mono‐organoid culture. Thus, the liver‐islet organoid system enabled effective maintenance of glucose levels within the normoglycemic range, reflecting the in vivo homeostatic feedback loop. However, the rates of insulin secretion and glucose uptake in the multi‐organoid system were slower than that seen in physiological conditions. This may be attributed to the absence of major glucose‐consuming periapical tissues, such as muscle, brain, and kidney.

Hyperglycemia is a major factor in the development of diabetes, leading to systemic pathological changes in many organs in patients. To mimic the glucose‐induced pathological changes in islet and liver cells, the islet‐liver organoid system was exposed to a high glucose medium. We then assessed mitochondrial function and observed reduced cellular OCR by directly quantifying mitochondrial respiration in the organoids. These results are consistent with previous reports that suggested that hyperglycemia and T2DM can induce mitochondrial dysfunction, ROS production, oxidative stress, and tissue damage.^[^ ^58^, ^59^ ^]^ The co‐cultured organoids demonstrated mitochondrial dysfunction and decreased expression of glucose transporter proteins following glucose stimulation (25 mm), which is consistent with other previous in vivo studies.^[^ ^58^, ^59^, ^60^ ^]^ Additionally, these pathological changes could be alleviated by the addition of metformin, a drug widely used to treat diabetes, indicating the capability of this liver‐islet organoid system to model the key pathological features of T2DM.

In summary, we provide a proof‐of‐concept to recreate the cooperative multi‐organoid system for modeling human‐relevant liver‐pancreatic islet axis in normal and type 2 diabetes conditions. The obvious advantage of this system lies in its ability to mimic human‐relevant functional coupling of the liver and islet organs response to external hyperglycemic stimulus and drugs, which are not easily studied by monolayer cell cultures and animal models. In future, this multi‐organoid system could be possibly integrated with other organs, such as brain, muscle, and fat, to reflect the systemic physiology and pathology in the human body. Additionally, this platform could be used to unravel the mechanisms and comorbidities associated with metabolic disorders, including NAFLD, hepatic insulin resistance, and steatohepatitis.

## 4 Experimental Section

### Design and Fabrication of the Microfluidic Chip Device

The multi‐organoid‐on‐chip device was designed and fabricated using standard soft lithography and micro‐molding methods.^[^ ^61^ ^]^ The chip device included two chambers (height: 800 µm, width: 1.5 mm, length: 1.75 mm), each chamber with 240 micro‐wells (diameter: 500 µm, depth: 800 µm) and an arrangement of 15 in lines and 16 in rows. The culture compartments were connected by micro‐channels (height: 100 µm, width: 100 µm, length: 250 µm). The chip was made of PDMS (Sylgard 184, Dow Corning, U.S.). A mixture of PDMS and the curing agent (10:1 w/w) was poured onto the two SU‐8 masters and degassed under a vacuum, then cured at 80 °C for 1 h in an oven. Finally, the device was immersed in water and sterilized in an autoclave.

### hiPSC Culture

Three kinds of human skin fibroblasts derived iPSC line were used and all of them were kindly given as a present from Dr. Ning Sun. The human iPSC line was maintained in mTeSR1 medium (Stemcell Technologies) on six‐well plates (Jet Biofil) precoated with Matrigel (1:50 dilution, BD). When the cells reached ≈80% confluence, the iPSC cells were digested with Accutase (Sigma) for continuous passage culture.

### Differentiation of Liver and Islet Organoids

In this work, liver and pancreatic islet differentiation protocols were used to generate organoids from hiPSCs as in the previous studies^[^ ^29^, ^62^ ^]^ with minor modifications. Briefly, an iPSC suspension was seeded on the micro‐well device (200 µm, diameter) for EB formation. These EBs were cultured in mTeSR1 medium with 10 µm Y27632 (Selleck) for 3 days. According to in vivo organ development processes, the liver and pancreas share a common endodermal origin during organogenesis.^[^ ^39^ ^]^ As such, they could be cultured in the same media at the DE differentiation stage. After EB formation, the culture medium was replaced with DMEM/F12 (Invitrogen) with 1% Knockout Serum Replacement (KSR, Invitrogen), 1% B27 supplement (Invitrogen), 50 ng mL^−1^ activin A (PeproTech), 1% GlutaMAX (Invitrogen), and 1% penicillin‐streptomycin (Invitrogen) for 5 days. Specific differentiation to pancreatic or hepatic lineages was then carried out on the micro‐well chip.

For the HP differentiation, the media was replaced with RPMI‐1640 medium (Invitrogen) supplemented with 1% KSR (Invitrogen), 1% GlutaMAX (Invitrogen), 1% B27 supplement (Invitrogen), 1% penicillin–streptomycin (Invitrogen), 100 ng mL^−1^ activin A (PeproTech), 10 ng mL^−1^ basic fibroblast growth factor (bFGF) (PeproTech), and 20 ng mL^−1^ HC growth factor (HGF, PeproTech). For HC differentiation and expansion, the media was replaced with HC Culture Medium (HCM, ScienCell) supplemented with 10 ng mL^−1^ oncostatin M (OSM, R&D) and 10^−7^ m dexamethasone (Sigma). The spheroids were then cultured in HCM medium with 10^−7^ m dexamethasone to facilitate further HC maturation.

For PE differentiation, cells were cultured in DMEM (Invitrogen) mixed with 0.5% B27 supplement (Invitrogen), 2 µm dorsomorphin (Selleck), 2 µm retinoic acid (RA, Sigma), 10 µm SB431542 (Selleck), 5 ng mL^−1^ bFGF (PeproTech), and 250 nm SANT‐1 (Selleck) for 6 days. Then, for EP cell differentiation, the medium was changed to DMEM mixed with 0.5% B27 supplement (Invitrogen), 2 µm dorsomorphin (Selleck), 10 µm SB431542 (Selleck), 50 µg mL^−1^ ascorbic acid (Sigma), and 10 µm DAPT (Abcam) for 4 days. Last, for EC differentiation, the media was replaced with CMRL 1066 (Invitrogen) containing 25 mm glucose (Sigma), 0.5% B27 supplement (Invitrogen), 10 mm nicotinamide (Sigma), 10 µm SB431542 (Selleck), 50 µg mL^−1^ ascorbic acid (Sigma), 2 µm dorsomorphin (Selleck), and 0.5% penicillin–streptomycin (Invitrogen) for 8 days.

### Construction of Multi‐Organoid‐On‐Chip System

To co‐culture the liver and islet organoids on the microfluidic chip system, the liver (150 spheroids, ≈10^7^cells) and islet (150 spheroids, ≈10^7^cells) organoids were collected and injected into the chip device through the liver and islet inlets, respectively. During the experiment, organoids were seeded in the chip through the cell inlet using a peptide gun and gently repeated several times until all of the organoids loaded into the micro‐wells (about 90% trap efficiency). The islet organoids dispersed and entered the microwells over a 10‐min period, and then the liver organoids were injected. Approximately 10 min later, the chip device was connected to peristaltic pumps, and fresh co‐culture media was pumped into the chambers. Two different flow rates (100 and 200 µL h^−1^) were tested in the experiment and selected the optimized condition (100 µL h^−1^) in the following experiment. Chip devices injected with liver or islet organoids (300 spheroids, ≈2 × 10^7^cells) alone served as the control groups. Defined co‐culture medium contained RPMI 1640 (Invitrogen) with 11 mm glucose, 0.5 m N‐acetylcysteine (R&D), 1% B27 supplement (Invitrogen), 1% N2 supplement (Invitrogen), 1% GlutaMAX (Invitrogen), 1% non‐essential amino acids (NEAA, Invitrogen), and 1% penicillin‐streptomycin (Invitrogen) and changed every two days.

### Immunohistochemistry

Liver and pancreatic islet organoids were collected by mechanically fracturing the PDMS chip device and then fixed in 4% paraformaldehyde for 30 min at room temperature. The fixed organoids were washed with PBS and dehydrated in a 30% sucrose solution at 4 °C overnight. The organoids were then embedded in an optimal cutting temperature compound (SAKURA) and stored at −80 °C. Cryosections (15 µm) were washed with PBS and permeabilized with 0.2% Triton X‐100 for 5 min. Then, the sections were blocked with goat serum (Solarbio) for 1 h and incubated with primary antibodies at 4 °C overnight. The primary antibodies used in each experiment are listed in Table 1. The samples were washed with PBS and then incubated with Cy3 (1:500, Beyotime) or Alexa Fluor 488‐ or 594‐conjugated secondary antibodies (1:500, CST) at room temperature for 1 h. Nuclei were stained with DAPI (1:4000, CST 4083).

### Real‐Time Quantitative PCR

To quantify the gene expression levels in liver and islet organoids, mRNA was isolated using Trizol reagent (TAKARA), and the final RNA concentration was adjusted to 125 ng µL^−1^. cDNA was synthesized via RT‐PCR according to the manufacturer's protocol using SYBR Green (TAKARA) under the following reaction conditions (40 cycles): denaturation at 95 °C for 1 min, annealing at 60 °C for 30 s, and extension at 72 °C for 30 s. The primer pairs used are listed in Table 2. The expression of *GAPDH* was used as a reference gene in each sample.

### Cell Viability Analysis

The cell viability of liver and islet organoids was assessed at different time points (0, 5, 15, and 30 days) during the co‐culture period using calcein‐AM and ethidium homodimer‐1 (LIVE/DEAD Viability/Cytotoxicity Assay Kit, Gibco, L3224). The procedure was performed according to the manufacturer's instructions.

### Analyses of Supernatant Samples

To compare ALB and insulin secretion from the liver and islet organoids, respectively, in the mono‐ and co‐culture group, medium was collected from the liquid storage tank in chip system every 5 days over a period of 40 days and stored at −80 °C. Collected supernatant samples were analyzed using the human albumin enzyme‐linked immunosorbent assay (ELISA) kit (Abebio, AE64523HU) and human insulin ELISA assay kit (Abebio, AE63173HU).

Glucose consumption and insulin secretion following the glucose‐stimulation test were also quantified. Several parallel chip devices were used to collect media samples every 6 h on days 1, 15, and 30. Samples were stored at −80 °C. The glucose concentration was measured using EnzyChrom Glucose Assay Kit (BioAssay Systems, EBGL) and insulin was quantified using an ELISA kit.

### UID (Unique Identifier) RNA Sequencing

The mono‐ and co‐cultured liver and islet organoids were collected on day 15, and total RNAs were extracted from samples using Trizol (Invitrogen) according to Chomczynski's study.^[^ ^63^ ^]^ Stranded RNA sequencing library preparation was carried out with 2 µg total RNAs and the KC‐Digital TM Stranded mRNA Library Prep Kit for Illumina (DR08502, Seqhealth) following the manufacturer's instructions. The sequencing data were then used for standard RNA‐seq analysis. Sequences were aligned to a *Homo sapiens* reference genome from Homoserines (GRCh38) using STAR software (version 2.5.3a) with default parameters. Alternative splicing events were detected using rMATS (version 3.2.5) with an FDR value cutoff of 0.05 and an absolute value of Δ*ψ* of 0.05. The RNA‐seq data reported in this paper were accessible in the SRA with the accession code PRJNA730722.

### Measurement of Oxygen Consumption Rate

To assess OCR in the co‐cultured liver and islet organoids in hyperglycemic (25 mm glucose) conditions, the liver and islet organoids were co‐cultured on the multi‐organoid‐on‐chip system for 15 days. Since then, they were exposed to co‐culture medium with no glucose, high glucose (25 mm glucose), or metformin (25 mm glucose and 100 mm metformin). The organoids were collected by mechanically fracturing the chip device. Basal OCR values were measured using an XF24 extracellular flux analyzer (Seahorse Bioscience, MA) according to the manufacturer's protocol and the data were normalized to the total cell number

### Statistical Analysis

Data were expressed as the mean ± standard deviation (SD) for three independent experiments. The difference between two groups was analyzed using Student's t‐test. Multiple group comparison was performed using one‐way analysis of variance (ANOVA) followed by post‐hoc test. Significance was indicated by asterisks: *p < 0.05; **p < 0.01; ***p < 0.001.

## Conflict of Interest

The authors declare no conflict of interest.

## Supporting information

Supporting Information

## Data Availability

The data that support the findings of this study are available from the corresponding author upon reasonable request.
