# Supplementary material for: Microengineered Multi‐Organoid System from hiPSCs to Recapitulate Human Liver‐Islet Axis in Normal and Type 2 Diabetes
Source: Adv Sci (Weinh). 2021 Dec 23;9(5):2103495. doi: 10.1002/advs.202103495 (PMC8844474; doi:10.1002/advs.202103495)
Supplement: Supplementary file 1 — Supporting Information [file ADVS-9-2103495-s001.pdf]

ADVANCED SCIENCE
Open Access

Supporting Information

for *Adv. Sci.*, DOI: 10.1002/advs.202103495

Microengineered multi-organoid system from hiPSCs to recapitulate human liver-islet axis in normal and type 2 diabetes

*Tingting Tao, Pengwei Deng, Yaqing Wang, Xu Zhang, Yaqiong Guo, Wenwen Chen, Jianhua Qin**

Supplementary Materials

Microengineered multi-organoid system from hiPSCs to recapitulate human liver-islet axis in normal and type 2 diabetes

Tingting Tao[1, 4], Pengwei Deng[1, 4], Yaqing Wang[1], Xu Zhang[1], Yaqiong Guo[1, 4], Wenwen Chen[1, 4], Jianhua Qin*[1, 2, 3, 4]

1 Division of Biotechnology, Dalian Institute of Chemical Physics, Chinese Academy of Sciences

2 Institute for Stem Cell and Regeneration, Chinese Academy of Sciences, Beijing, China.

3 CAS Center for Excellence in Brain Science and Intelligence Technology, Chinese Academy of Sciences, Shanghai, China.

4 University of Chinese Academy of Sciences, Beijing, China.

*Corresponding Author. Jianhua Qin, Dalian Institute of Chemical Physics, Chinese Academy of Sciences, 457 Zhongshan Road, Dalian 116023, China. E-mail: jhqin@dicp.ac.cn (J.Q.).

Supplementary Figures

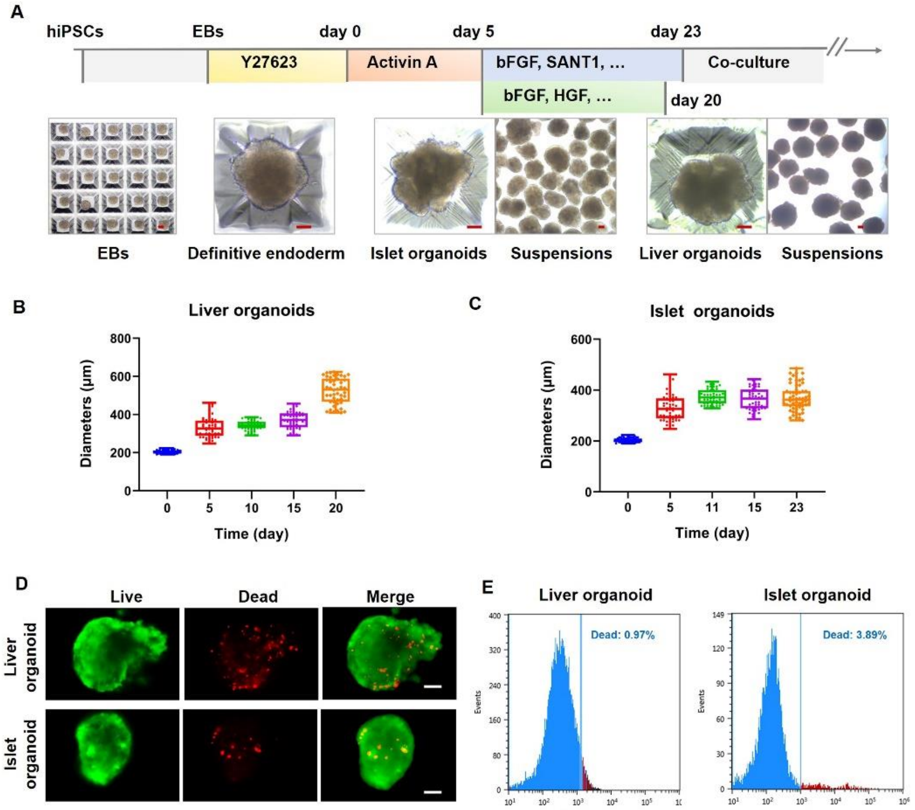

Supplementary Fig. 1. Generation and development of liver and islet organoids derived from hiPSCs. (A) Bright-field images of liver and islet organoids at different stages during the differentiation period. (B–C) Organoid morphology was analyzed using the average diameters of the liver and islet organoids at different time points. (D–E) Live/dead and flow cytometry assays were conducted to assess the cell viability of organoids by staining with a live/dead kit on days 20 and 23. Scale bars: 100 μm.

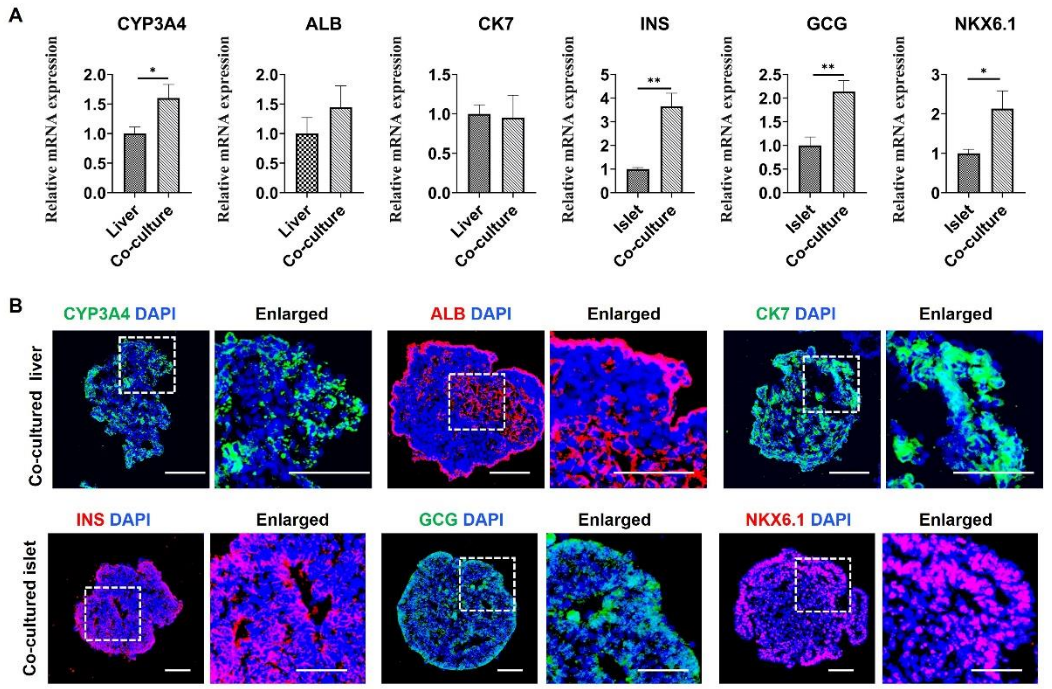

Supplementary Fig. 2. The expression of functional proteins and mRNAs in liver and islet organoids under mono- and co-culture conditions on day 5. (A) Quantification of the relative mRNA expression levels of liver-associated genes (*CYP3A4*, *ALB*, and *CK7*) in liver organoids and islet-associated genes (*NKX6.1*, *INS*, and *GCG*) in islet organoids using RT-PCR. Expression values were normalized to GAPDH. Relative expression is represented as the mean ± SEM, three independent experiments were performed. The data were analyzed using Student's t-test, *$p < 0.05$, **$p < 0.01$, and ***$p < 0.001$. (B) Immunohistochemical staining of CYP3A4 (red), ALB (red), and CK7 (green) in liver organoids and NKX6.1 (red), INS (red), and GCG (green) in pancreatic islet organoids. DAPI was used to stain the nuclei (blue). Scale bars: 100 μm. Enlarged views are shown to the right of each image. Scale bars: 20 μm.

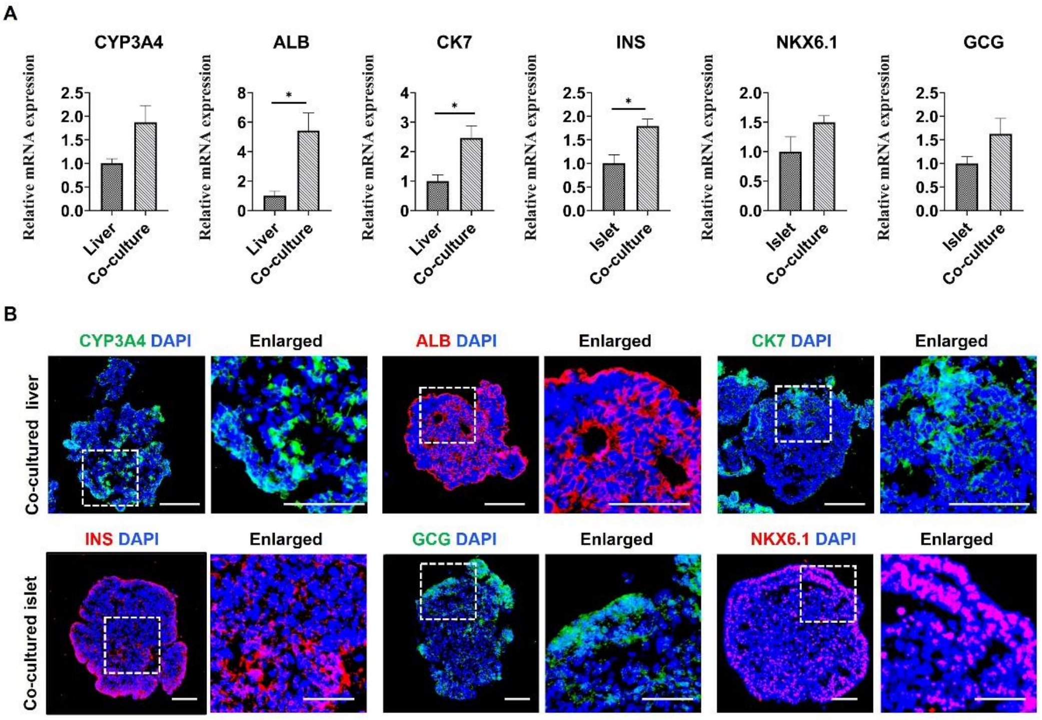

Supplementary Fig. 3. Comparison of the expression of tissue-specific proteins and mRNA in liver and islet organoids under mono- and co-culture conditions on day 30. (A) Quantification of relative mRNA expression levels of liver-associated genes (*CYP3A4*, *ALB*, and *CK7*) and islet-associated genes (*NKX6.1*, *INS*, and *GCG*) in organoids using RT-PCR. Expression values were normalized to GAPDH. Relative expression is represented as the mean ± SEM, three independent experiments were performed. The data were analyzed using Student's t-test, $*p < 0.05$, $**p < 0.01$, and $***p < 0.001$. (B) Hepatic-specific markers CYP3A4 (red), ALB (red), and CK7 (green) in liver organoids and pancreatic-associated markers NKX6.1 (red), INS (red), and GCG (green) in islet organoids were identified by immunofluorescence staining. DAPI was used to stain the nuclei (blue). Scale bars: 100 µm. Enlarged views are shown at the right side. Scale bars: 20 µm.

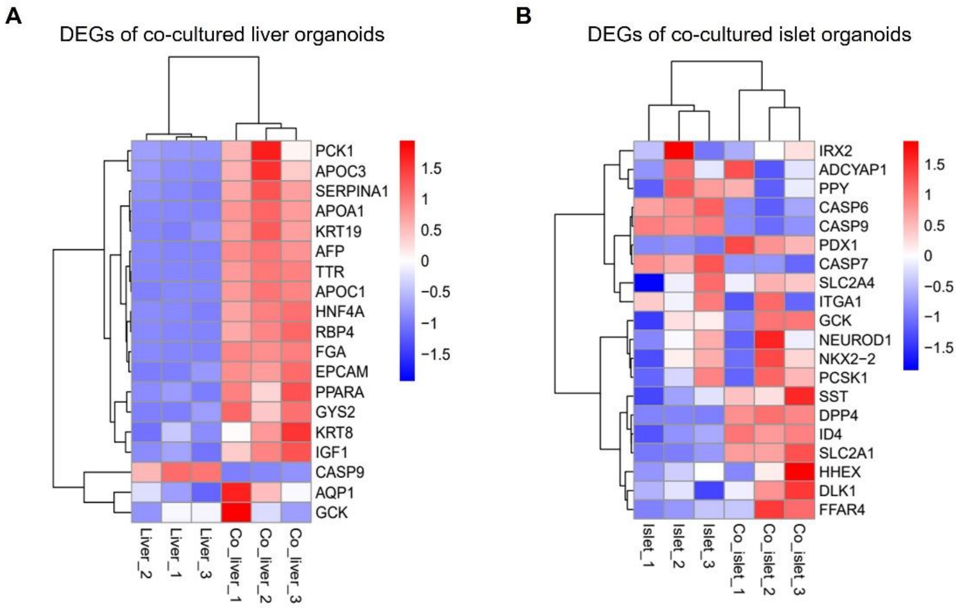

Supplementary Fig. 4. Analysis of functional DEGs in liver and islet organoids in the multi-organoid-on-chip system. (A-B) Heat map of the liver and islet organoid DEGs under mono- and co-culture conditions at day 15. Three repeats were performed. The gradient color scale at the right top indicates the $\log_2$ (fold change) in expression of the co-cultures compared with the mono-cultures.

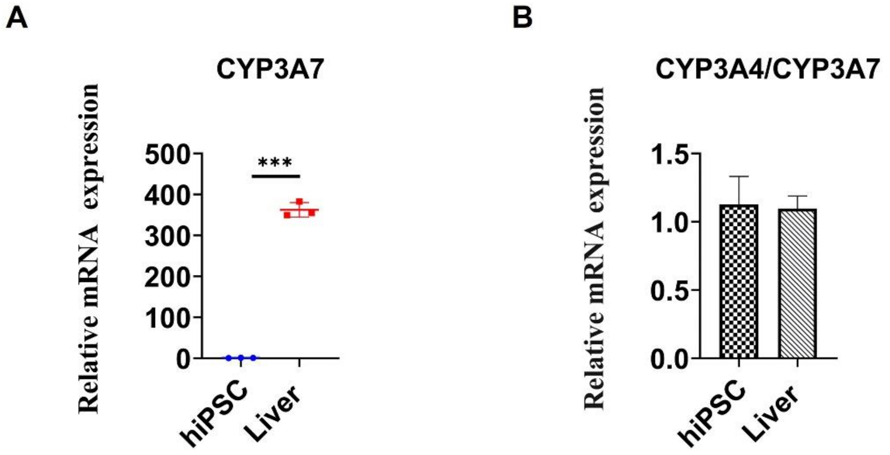

Supplementary Fig. 5. mRNA expression levels of CYP3A7 and CYP3A4/CYP3A7 ratios in the hiPSCs derived liver organoids. (A) Quantification of relative mRNA expression levels of liver-associated genes *CYP3A7* in liver organoids using RT-PCR. (B) *CYP3A4/CYP3A7* mRNA expression ratios in the liver organoids. Expression values were normalized to GAPDH. Relative expression is represented as the mean ± SEM, three independent experiments were performed. The data were analyzed using Student's t-test, ***$p < 0.001$.

Supplementary Tables

Supplementary Table 1. Primary antibody information

Target	Dilution	Host	Company
INS	1:1000	guinea pig	Abcam, ab7842
SST	1:100	rat	Abcam, ab30788
PPY	1:100	rabbit	Gene Tex, GTX128056
GCG	1:100	rabbit	ThermoFisher, PA513442
NKX6.1	1:500	rabbit	CST, D804R
ALB	1:1000	goat	Bethyl, A80-129A
CYP3A4	1:500	mouse	Absin, abs132219
CK7	1:8000	rabbit	Abcam, ab181598
GLUT1	1:500	rabbit	RuiYing Bio, RLT1928
GLUT4	1:1000	rabbit	Abcam, ab654

Supplementary Table 2. RT-PCR primer sequences

Primer	Forward sequence (5′→3')	Reverse sequence (5'→3')
INS	TGTACCAGCATCTGCTCCCTCTA	TGCTGGTTCAAGGGCTTTATTCCA
SST	CCCCAGACTCCGTCAGTTTC	TCCGTCTGGTTGGGTTCAG
PPY	ACCTGCGTGGCTCTGTTACT	TACCTAGGCCTGGTCAGCAT
GCG	AGGCAGACCCACTCAGTGA	AACAATGGCGACCTCTTCTG
NKX6.1	ATTCGTTGGGGATGACAGAG	TGGGATCCAGAGGCTTATTG
ALB	GCCTTTGCTCAGTATCTT	AGGTTTGGGTTGTCATCT
CYP3A4	TTCAGCAAGAAGAACAAGGACAA	GGTTGAAGAAGTCCTCCTAAGC
CYP3A7	AAACTTGGCCGTGGAAACCT	CAGCATAGGCTGTTGACAGTC
CK7	AAGAACCAGCGTGCCAAGTT	CACGCTCATGAGTTCCTGGT
SLC2A1	GCAACGGCTTAGACTTCGAC	TGCGACTTCAGGCACATAAC
SLC2A4	TGTCACAGTCCCCAACACCA	CCGAAGCATGTGGAAAGCA
